# Connected in diversity: Isotopic analysis refines provenance for Islamic plant-ash glass from the eastern Silk Roads

**DOI:** 10.1016/j.isci.2023.108450

**Published:** 2023-11-14

**Authors:** Qin-Qin Lü, Hassan Basafa, Julian Henderson

**Affiliations:** 1 Department for the History of Science and Scientific Archaeology, University of Science and Technology of China, Hefei 230026, China; 2 McDonald Institute for Archaeological Research, University of Cambridge, Cambridge CB2 3ER, UK; 3 St. Edmund’s College, University of Cambridge, Cambridge CB3 0BN, UK; 4 Department of Archaeology, University of Neyshabur, Neyshabur 9319774446, Iran; 5 Department of Classics and Archaeology, University of Nottingham, University Park, Nottingham NG7 2RD, UK

**Keywords:** Earth sciences, Social sciences, Archeology

## Abstract

Our understanding of glass production in Eurasia has been built mostly on evidence from Europe and the Mediterranean. Here, we investigate the occurrence and organization of plant-ash glass production in the eastern continental Islamic region, focusing on an 11^th^–12^th^ century assemblage unearthed in Shadyakh, Nishapur, Iran. Through Sr-Nd isotope analysis and by examining geochemical contexts and mixing patterns, we find that distinct silica and ash sources originating from Tigris-Euphrates Basin, Central Asia, and potentially Iran were used to make these objects. Zagros-derived silica and Central-Asian-type silica were likely important silica sources for Islamic plant-ash glasses from east of the Tigris. Furthermore, we show that Central Asian glass can be characterized by chemical and isotopic signatures, while Iranian glass may exhibit overlapping signatures with glass from neighboring regions. The plant-ash glass industry in Islamic-period West and Central Asia likely thrived by exploiting and sharing diverse, regionally characteristic raw material sources.

## Highlights

• Sr-Nd isotopes help characterize diverse plant-ash glasses from eastern Silk Roads
• Decentralized production likely used diverse raw materials within regional networks
• Trade brought glass from Iraq, Syria, Central Asia, and potentially Iran to Shadyakh
• Regional signatures distinguish between Zagros-derived and Central-Asian-type silicas

## Graphical abstract

**Figure:**
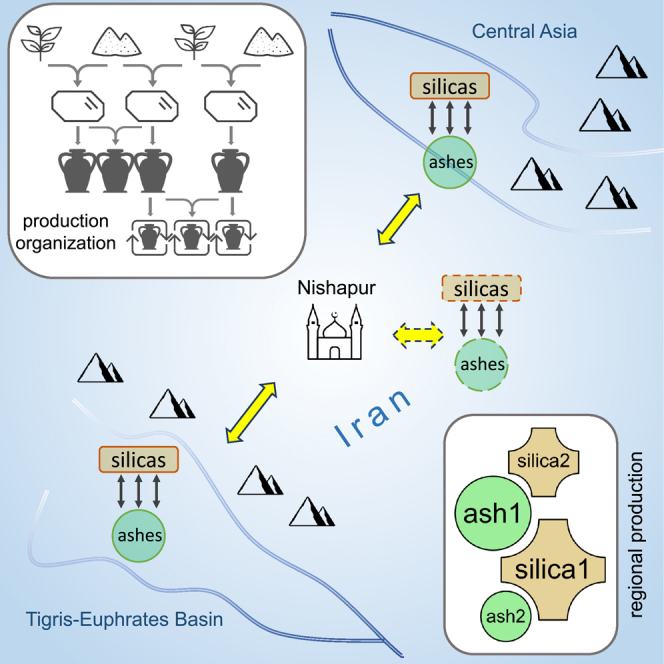

## Introduction

Scientific investigation of archaeological materials can inform us about the utilization of natural resources, knowledge generation and transmission, and socioeconomic relations in past societies. One of the most widely used synthetic materials in history, glass was extensively traded along the Silk Roads, a transcontinental network of communications (e.g.^1^^,^^2^^,^^3^^,^^4^). In particular, Islamic plant-ash glass (circa 9^th^–15^th^ century) offers insights into the trade and technological evolution along the medieval Silk Roads (e.g.^5^^,^^6^^,^^7^).

Provenancing glass artifacts to their sources is important to understand glass trade. Recent archaeometric research, mainly based on chemical analysis and particularly trace elements, has identified compositional groups of Islamic plant-ash glass associated with production zones in Egypt, the Levant, and the Tigris-Euphrates River Basin.^8^^,^^9^^,^^10^^,^^11^^,^^12^^,^^13^^,^^14^^,^^15^^,^^16^^,^^17^^,^^18^ Plant-ash glass was made mainly using geological (quartz sands or crushed pebbles, providing silica) and botanical (ashed halophytic plants, providing soda) ingredients. The occurrence of primary glassmaking is supported by raw furnace glass, which has been found in early Islamic sites such as Tyre (Lebanon)^12^^,^^19^ and Raqqa (Syria).^18^^,^^20^^,^^21^^,^^22^ Secondary production evidence has been widely reported, e.g., in Banias^16^ and Fustat.^9^

The introduction of strontium (Sr) and neodymium (Nd) isotope analyses has proven useful for plant-ash glass provenance. Sr is mostly introduced by the lime content in plants, reflecting the bioavailable Sr pool of the environment, which is controlled by the local geology and environment. Nd is mainly derived from the non-quartz minerals in regional clastic sediments used as vitrifying materials. The Sr and Nd isotopes function as unique markers for the origins of plant ash and silica, respectively, allowing the two main glassmaking ingredients to be traced separately.^13^^,^^17^^,^^23^^,^^24^^,^^25^^,^^26^^,^^27^^,^^28^ A combination of chemical and Sr-Nd isotopic compositions may shed light on glass manufacturing processes such as production recipes and raw material supplies.

One of the main hindrances to a better understanding of Islamic plant-ash glass is a research imbalance between western and eastern Islamic glasses. Compared to the western regions (e.g., the Levant, Egypt, and Syria), Islamic plant-ash glasses from Iran and Central Asia remain understudied: the status of glass production is unclear, and how glass production was organized is not understood. Almost no glassmaking evidence has been reported other than the preliminary analysis of furnace glass in Akhsiket (Uzbekistan).^29^ Moreover, only a small portion of Islamic glass discovered in Iran and Central Asia has been analyzed (e.g.^11^^,^^29^^,^^30^^,^^31^^,^^32^^,^^33^^,^^34^^,^^35^^,^^36^^,^^37^), many of which are museum objects, surface finds, or from early excavations without clear contexts or unbalanced collections skewed toward high-value objects. Notably, few isotopic data for Islamic plant-ash glass have been formally reported from regions to the east of Syria, except for the pioneering Sr isotope analyses of a limited number of samples^25^^,^^38^ (see Tables S3–S5, in the Supplementary Material). To our knowledge, no Nd isotopic data are available for Iranian and Central Asian glasses.

In this study, we focus on Islamic plant-ash glass from Iran and Central Asia through a case study on archaeologically excavated vessel fragments from a workshop in Shadyakh, Nishapur, northeastern Iran. Using chemical elements and Sr-Nd isotopes, we establish technological links with other assemblages in West and Central Asia and associate the glass with various sources of plant ash and silica, which helps to understand glass trade and the organization of glass production in the eastern continental Islamic regions.

Nishapur, located on the Silk Road route connecting Iran and Central Asia, was a major trade and political center of the Greater Khorasan region which encompasses northeastern Iran, northern Afghanistan, and Turkmenistan. It was founded in the early period of Sasanian Dynasty (3^rd^–7^th^ century), rose to prominence in the 10^th^ century under Samanid rule, and briefly served as the capital of the emerging Seljuk Empire in the 11^th^ century. The city’s prosperity declined in the 12^th^ century until its destruction during the Mongol invasion in 1221 CE. Geologically, Nishapur is situated on the southern slope of Binalud Mountain, an extension of the eastern Alborz range. Paleozoic to Cenozoic formations dominate the Nishapur Plain and the Binalud Mountain.^39^ Cenozoic fluvial sediments from small river systems originating from the Binalud are present near Nishapur.^39^^,^^40^ A large reserve of quartz pebbles was found near Mashhad in the exposed Middle Jurassic formation.^41^

The archaeological investigation of Nishapur began with the Metropolitan Museum of Art’s (the Met) excavations in the 1930s–1940s, which uncovered large quantities of glass artifacts, although mostly without stratigraphic information.^42^ Coins and ceramic finds indicate a 9^th^–10^th^ century date.^30^ The glass finds included a variety of vessel forms, such as bowls, plates, beakers, jugs, and bottles, and most of the glasses were colorless or close to colorless. Both free-blown and mold-blown vessels were reported, and the decorations involved wheel-cutting, applying, incising, pinching, and stamping techniques.^42^ Although a number of glass slabs or ingots were found, only two glass chunks of green or blue color survived. No other evidence of glass manufacturing was discovered.

**Figure 1:**
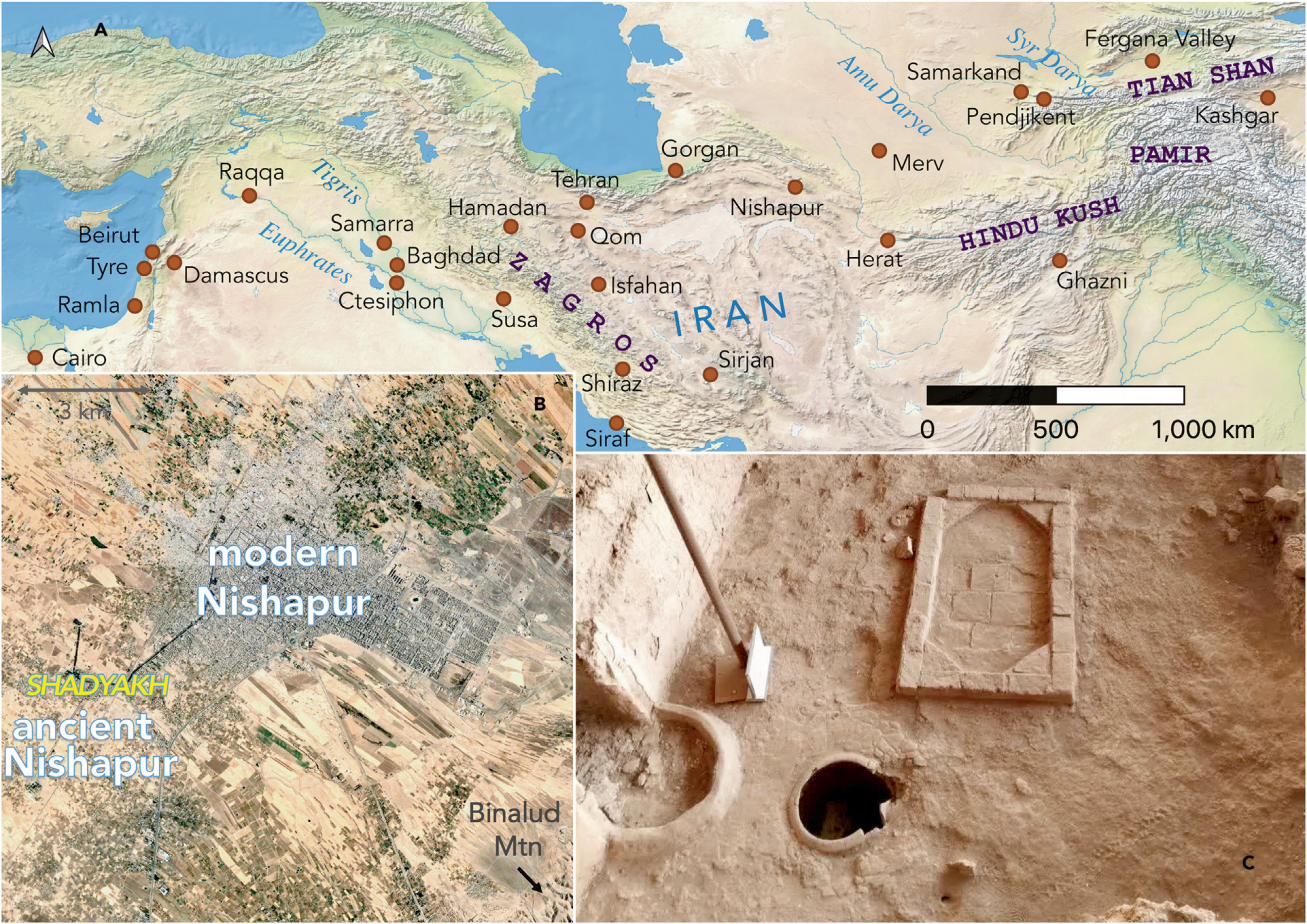
(Color online): Geographical context of the samples (A) Nishapur’s location in West and Central Asia. The map was created with QGIS (https://www.qgis.org/) with the *Natural Earth II* layer. (B) Shadyakh’s location in Nishapur. The image is based on Google Earth satellite map. (C) A possible glass workshop in Trench A-II. The photo is used with permission by Mr. Rajab-Ali Labaf Khaniki.

Shadyakh, meaning “the Palace of Happiness”, was an affluent, well-documented urban center of Nishapur.^43^ Shadyakh was established by the Tahirids and reached its peak in the Seljuk period (1040–1157 CE). As one of the main districts of Nishapur, Shadyakh was complete with palaces, houses, mosques, and cemeteries. The Met’s excavation identified Shadyakh in the west of ancient Nishapur. From 1999 to 2005, seven seasons of excavation were conducted at Shadyakh by Iranian archaeologists under the direction of Rajab-Ali Labaf Khaniki, which revealed that Shadyakh was a quadrangular district surrounded by a rampart. The excavations also uncovered buildings, residential and industrial zones, and an irrigation system.^44^^,^^45^ The location of Shadyakh in relation to ancient and modern Nishapur is shown in Figure 1.

**Figure 2:**
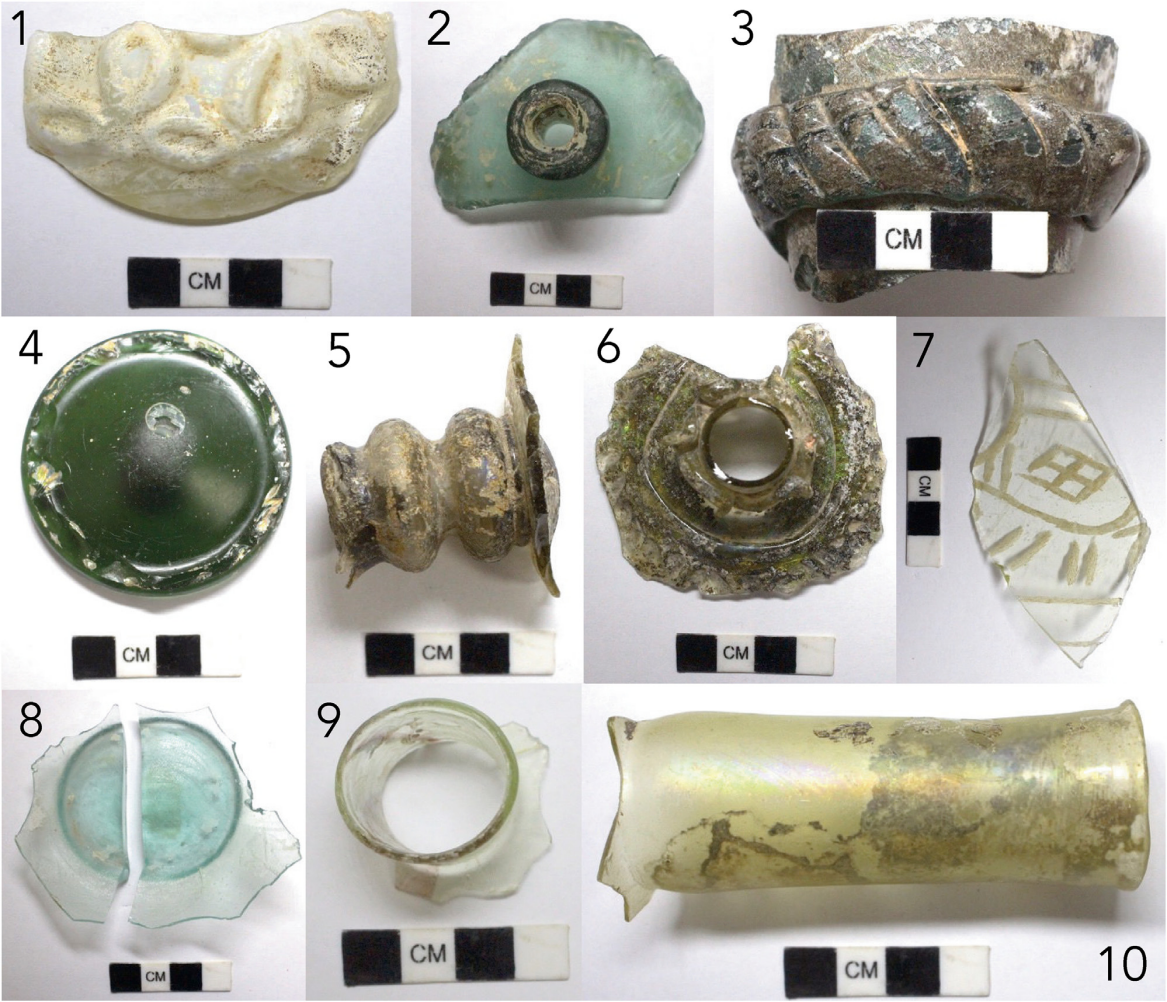
(Color online): The analyzed glass samples (The panel numbers indicate samples SDK1–SDK10)

Excavations in Shadyakh recovered possible glass workshops. Rajab-Ali Labaf Khaniki’s team found a large number of glass fragments, plaster molds, and slags on the porch of a building. On the south side of Trench A-I, a furnace with ash inside was discovered. The hemispherical furnace has a diameter of approximately 1.5 m and a height of less than 1 m. Burnt soil and possible slag were present. Pieces of melted glass were found on the furnace floor. Materials and waste of unknown nature were discovered in front of the furnace. In the southwest corner of Trench A-II in the industrial zone in the center of Shadyakh, in the middle of a brick-paved courtyard, a brick frame approximately 2 by 1 m with a plastered inner surface was found, and burn marks were visible on the bricks (Figure 1C; also see Figures S1 and S2 in the Supplementary Material). It was proposed that the frame’s floor was used for glassworking.^44^^,^^45^ The samples (Figure 2) were excavated as fragments from a depth of 2 m from this putative workshop in Trench A-II. They were dated to the 11^th^–12^th^ century in the Seljuk period based on an archaeological assessment of architecture, pottery, and other artifacts. The samples exhibit a diverse range of colors and forms, representing utilitarian glass in Nishapur. A detailed sample description, including inferred vessel forms, is given in the Materials section.

## Results and discussion

### Data

The chemical composition (Table S1) was obtained using Laser Ablation Inductively Coupled Plasma Mass Spectrometry (LA-ICP-MS), and the Sr-Nd-Pb isotopic compositions (Table S2) were determined using Multi-Collector Inductively Coupled Plasma Mass Spectrometry (MC-ICP-MS). A complete description of the experimental procedures is given in the method details section. SDK9 was not sampled for isotopic analysis since it is too thin and visibly corroded. The Sr isotopes are reported using the ^87^Sr/^86^Sr ratio, while the Nd isotopes are indicated using the ε_Nd_ notation

$$\varepsilon_{\mathrm{Nd}}=\left[\frac{{\left({\mathrm{Nd}}_{143}/{\mathrm{Nd}}_{144}\right)}_{\mathrm{sample}}}{{\left({\mathrm{Nd}}_{143}/{\mathrm{Nd}}_{144}\right)}_{\mathrm{CHUR}}}-1\right]\times 10^{4}$$

where (^143^Nd/^144^Nd)_CHUR_ = 0.512638. ε_Nd_ describes the sample’s Nd isotope ratio in terms of deviation from the present-day value of the Chondritic Uniform Reservoir (CHUR).^46^ The lead isotopes are reported as the ^206^Pb/^204^Pb, ^207^Pb/^204^Pb, and ^208^Pb/^204^Pb ratios.

### Chemical analysis

**Figure 3:**
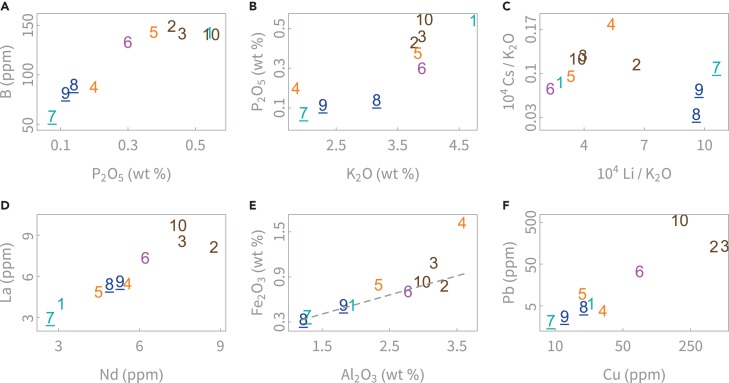
(Color online): Bivariate chemical relations indicate diverse sources of raw materials (A–F) show unique plant ash for SDK7, 8, and 9 (underlined) and five tentative types of silica (color-coded: SDK2, 3, 10; SDK1, 7; SDK 8, 9; SDK4, 5; SDK6). These groupings are not final results. See Table 1 for proposed origins for these objects.

**Figure 4:**
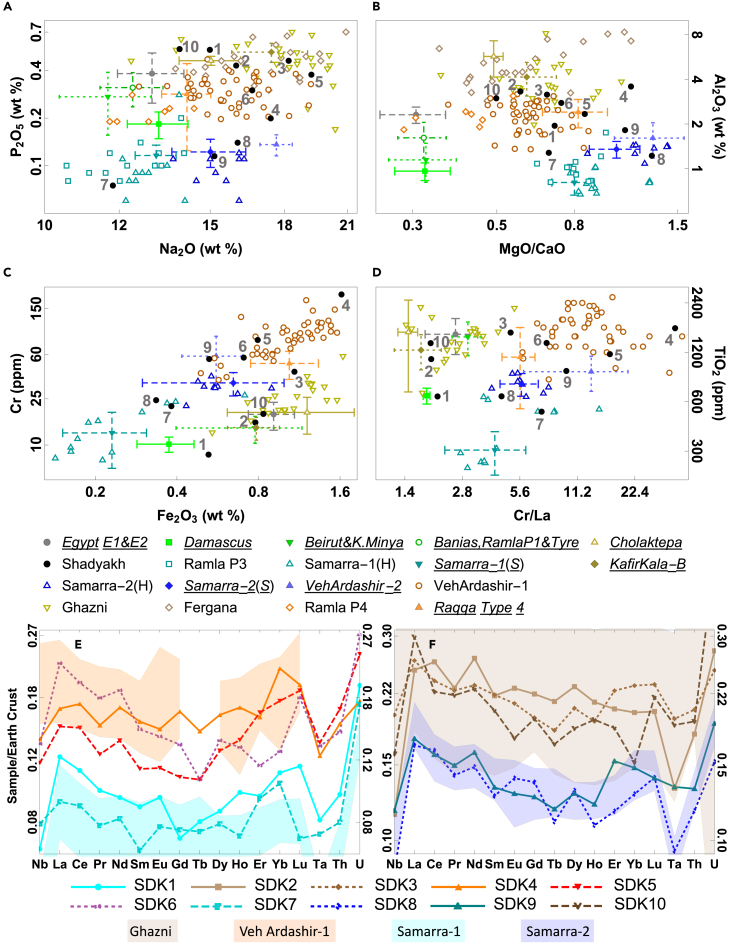
(Color online): Chemical relations of Shadyakh (SDK1–10) samples and Islamic and Sasanian plant-ash glasses from other locations The data include (1) Egyptian weights (Groups E1 and E2)^8^; (2) glass from the Levant, including artifacts from Beirut, Khirbat al-Minya, Damascus, and Ramla^11^^,^^15^ and predominantly raw glass from Tyre and Banias^12^^,^^16^; (3) glass from Samarra (referred to with H or S for the lead author of the data source) and Raqqa (only the most relevant subgroup—Raqqa Type 4)^10^^,^^11^^,^^18^; and (4) glass from Ghazni, Afghanistan,^31^ Akhsiket and Kuva, Fergana Valley,^29^ and Cholaktepa and Kafir Kala near Samarkand (only Group B).^33^ Sasanian plant-ash glass from Veh Ardashir^50^^,^^51^ is also included. Not all elements were reported for every sample. The *underlined italicized* assemblages in the legend for (A–D) are displayed as average values plus/minus one standard deviation (for Raqqa Type 4, the plotted range is half of the standard deviation). Element concentrations in (E) and (F) are normalized to the upper continental crust compositions.^52^

In plant-ash glass, the chemical elements generally attributed to plant ash are Na, P, Mg, Ca, B, K, Rb, Cs, and Li, while Al, Fe, Zr, Ti, Cr, and rare earth elements (REEs) are mainly associated with the silica source.^15^^,^^47^^,^^48^ The actual situation may be more nuanced (see previous discussions^11^^,^^47^^,^^49^). Our interpretation follows the aforementioned hypothesis with adjustments where necessary. Chemical compositions within the Shadyakh assemblage are compared in Figure 3. Reported data from surrounding locations are compared in Figure 4.

The Shadyakh samples contain 1.8%–4.7% K_2_O and 4.4%–6.9% MgO, consistent with the use of plant ash as the soda source. With the exception of SDK7, the samples contain 60%–67% SiO_2_ and 14%–19% Na_2_O. SDK7, a scratch-decorated colorless sample, stands out with its high SiO_2_ and low Na_2_O contents, implying a very high fusing temperature. Al_2_O_3_ and CaO vary from 1.2% to 3.6% and 5.3% to 8.9%, respectively. SDK4, 8, and 9 are notable for their relatively high Mg levels. The deep green samples SDK4 and SDK3 exhibit the highest Fe_2_O_3_ levels (>1%), suggesting iron’s role in coloration. All the colorless samples (SDK1, 6, 7, 8, 9, 10) display higher Mn/Fe ratios (Table S1), with SDK6, 9, and 10 containing more than 1% MnO, indicating the role of Mn as a de-colorant. The addition of Mn may have also introduced Ba, e.g., for SDK10, as certain manganese ores are known to be rich in Ba.^48^^,^^53^

The data show a clear distinction between the high-B, high-P, high-K samples (SDK1, 10, 2, 3, 5, 6) and the low-B, low-P, low-K samples (SDK7, 9, 8, 4) (Figures 3A and 3B), suggesting that the two groups employed different ash sources, although within each group more than one source may have been utilized. SDK7, 9, and 8 likely shared a common soda source since they consistently form a cluster and display positive correlations in the P-B, K-P (also see K_2_O/P_2_O_5_ in Table S1), P-Na, and Cs/K vs. Li/K relations (Figures 3 and 4). To highlight this shared plant-ash source, SDK7, 9, and 8 are underlined in Figure 3.

In terms of impurities in silica sources, a low-impurity group (SDK7, 1, 8, 9) and a high-impurity group (SDK2, 3, 10, 6) are observed in the Nd-La and Al-Fe scatterplots (Figure 3), as also indicated by the levels of Al, Ti, and heavy elements (Figure 4). SDK1 and 7 contain very low levels of heavy elements, suggesting the use of clean quartz minerals such as crushed quartz pebbles, whereas SDK8 and 9 display slightly higher levels of heavy elements. SDK7, in particular, was likely produced using a high-quality silica material to achieve total transparency. SDK2, 3, and 10 are rich in heavy elements including REEs and were likely made using high-impurity sands. Compared to SDK2, 3, and 10, SDK6 displays similar Al and Ti levels, lower REE levels, and distinctly higher Zr and Hf levels. SDK4 and 5 contain comparable Al and Ti levels to SDK2, 3, 10, 6, and exhibit similar levels of light REEs to SDK8 and 9, but their heavy REE concentrations are higher. A tentative grouping of different silica sources is represented by colors in Figure 3: blue (SDK8, 9), cyan (SDK1, 7), orange (SDK4, 5), brown (SDK2, 3, 10), and purple (SDK6). Minor differences exist within the clusters (e.g., SDK2 has lower Zr, Ti, Nb, and Hf concentrations than SDK3 and 10; and SDK1 contains more Al and Fe contents than SDK7) with ramifications that are discussed later.

The high-Fe, high-Al SDK4 is noteworthy (Figure 3E). A positive correlation between Fe and Al is commonly encountered in quartz sands and may suggest a general level of naturally occurring impurities from the sands.^18^ SDK4 deviates from the Fe-Al line formed by other samples, indicating the addition of an iron-rich material or a unique high-Fe silica source. Additionally, the significant Cr level in SDK4 (Figure 4C) suggests a contribution of chromite to the iron content. SDK4’s Ni and Ti levels are also rather high. A high Cr content is consistent with Tigris-Euphrates Basin (TEB) glass and likely suggestive of influence from the Zagros-Taurus orogenic belt, since the ultramafic source rocks in the mountainous headwaters of the Tigris and Euphrates could have been eroded to produce chromite-bearing sands.^54^

The levels of Cu and Pb vary among these samples (Figure 3F), but neither is sufficiently high to affect the color. The average Cu and Pb abundances in the Earth’s upper crust are 25 ppm and 20 ppm, respectively.^55^ The Cu and Pb contents in SDK2, 3, and 10 significantly exceed the natural background. For natron glass, elevated levels of coloring or opacifying elements in glass not showing such colors may suggest the occurrence of glass recycling.^56^ This could similarly apply to mixed plant-ash glass (although the thresholds of these elements may differ). The levels of colorants, however, cannot identify the mixing between colorless or naturally colored glass. For Islamic plant-ash glass, it is known that recycling occurred, attested by cullet and broken vessels recovered from the Serçe Limani shipwreck^57^ and by Nd isotope mixing lines.^23^ It is plausible that SDK2, 3, and 10 were manufactured from melts that incorporated colored glass.

The Shadyakh samples were compared with contemporary Islamic plant-ash glass from the Middle East and Central Asia (Figure 4). To avoid confusion, the term “Tigris-Euphrates Basin” (TEB) is used instead of “Mesopotamia” to refer to the area approximately corresponding to present-day Iraq and eastern Syria, e.g., TEB glass, which can include Mesopotamian, Sasanian, or Islamic glass. “Mesopotamia” is retained for geological contexts (in line with Mesopotamian Floodplain and Foredeep) and when citing previous works (e.g., Mesopotamian Type, although we argue TEB Type may be more appropriate). In Figure 4, most samples from the Levant belong to the Levantine type^11^^,^^12^^,^^16^ except the imported vessels found in Ramla.^15^ Both the Egyptian and Levantine groups have low magnesia and high lime contents, leading to very low MgO/CaO ratios. They also display low Cr/La ratios.^11^^,^^54^ Two low-impurity groups (Samarra-1 and Samarra-2) have been identified in Samarra glass,^10^ which have been collectively referred to as Mesopotamian Type 2.^15^ Ramla P3 samples share similar compositions with Samarra-1. Mesopotamian Type 2 is characterized by high MgO/CaO ratios and generally low impurities (Figure 4). Meanwhile, the Ramla P4 group was referred to as Mesopotamian Type 1,^15^ but its levels of flux-related elements are lower than most TEB glasses. Veh Ardashir Group 1 and Raqqa Type 4 show some compositional similarities and could be related to Mesopotamian Type 1, although both are compositionally varied (glass mixing was suggested for Raqqa Type 4^18^). They are characterized by high Cr, Fe, and Ti levels and high Cr/La ratios.^15^^,^^32^ Their levels of P, B, Al, and heavy elements and MgO/CaO ratios are intermediate compared to other groups. The Cr-Fe relation may result from the relative abundance of (ultra)mafic/felsic source rocks for the vitrifying materials, and (ultra)mafic rocks typically contain higher levels of Cr and Fe than felsic rocks. The TEB glasses form a positive Cr-Fe correlation line. Beneath TEB glasses, Central Asian and Levantine samples show lower Cr and higher Fe levels (Figure 4C).

Glasses from Ghazni, the Samarkand region (Cholaktepa and Kafir Kala), and the Fergana Valley are grouped together as the Central Asian type (Figure 4). These glasses contain elevated levels of Na, P, and B, and the high flux content suggests a relatively low fusing temperature. The levels of Al, Ti, Fe, and heavy elements are consistently high, pointing to the use of impure silica sources. Distinctive characteristics of this type are the markedly low Cr levels and low Cr/La ratios. Part of Central Asian glass exhibits minor overlaps with Mesopotamian Type 1, e.g., in P, Al, and Ti concentrations.

While some Shadyakh samples align with specific glass types, others demonstrate ambiguous associations that require further scrutiny. At first glance, the REE levels of the low-impurity samples SDK1 and 7, both colorless, seem to match those of the low-impurity group Samarra-1 (Figure 4). The compositional similarity between Nishapur’s cut colorless vessels and Samarra glass has been noted previously,^11^^,^^32^ and the provenance for SDK7 may well be Samarra. However, unlike SDK7 with incised decoration, SDK1 is mold blown, and its ash composition differs drastically from SDK7 and is closer to Central Asian and certain TEB samples, as indicated by the P, K, B, and Cs levels. Also, compared to the Samarra-1 type, SDK1 has a lower Cr/La ratio and higher Al, Fe, and Ti levels (Figure 4), indicating that it might not be a Samarra product. SDK8 and 9 are clearly associated with Samarra-2. With higher impurities, SDK6 and SDK4 seem to correspond to Mesopotamian Type 1 or TEB glass. SDK4’s high Al level is likely due to the Fe- and Cr-rich material involved in its production (Figure 4B). SDK5 mostly aligns with TEB glass, but its P and B levels are comparable to those of Central Asian and Egyptian samples. Regarding the high-impurity samples SDK2, 3, and 10, their Cr-Fe relations and REE levels align with Central Asia. However, their P, Al, and Ti levels lie at the boundary between typical TEB and Central Asian ranges, making it difficult to suggest a definite provenance. Furthermore, SDK3’s Cr/La ratio also falls between the values of the two groups (Figure 4).

### Provenancing with Sr-Nd isotopes

Although early Islamic plant-ash glasses in the Levant, Syria, and Iraq exhibit regional distinctions in trace elements, there are uncertainties when attempting to provenance Islamic plant-ash glass from further east on the Silk Roads solely based on chemical compositions, as highlighted by the above analysis. The compositional variations can be attributed to diverse sources of ash and silica and compositional fluctuations in the raw materials used. The occurrence of glass recycling may further complicate the identification of compositional signatures.

**Figure 5:**
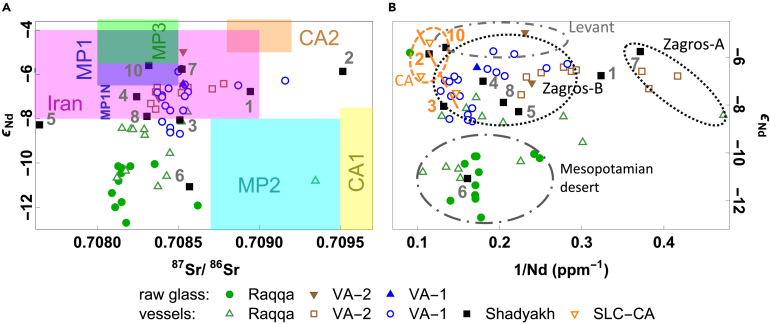
(Color online): Isotopic relations of Silk Road plant-ash glass The compared samples include Shadyakh (SDK1–10) samples and samples from Raqqa,^14^ Veh Ardashir (VA),^58^ and San Lorenzo (SLC).^59^ (A) Sr-Nd isotopic relation and (B) Nd isotopic-chemical relation. The isotopic zones are Mesopotamia/Tigris-Euphrates Basin—MP-1, MP-2, MP-3; Central Asia—CA1, CA2 (CA3 is beyond the plotted range); Iran.^23^

Recently, Lü et al.^23^ proposed an integrative approach that incorporates chemical composition, Sr and Nd isotopes, Nd isotope mixing lines, and Sr-Nd isotope baselines. Sr-Nd isotopes reveal potential sample connections through signatures of radiogenic isotopes, while the Nd isotopic-chemical relationship (mixing line) captures the dynamics of mixing processes (e.g., sediment mixing during transport, glass recycling). The baselines of bioavailable Sr and detrital Nd isotopes are ultimately controlled by regional geo-environmental conditions and serve as the geochemical context for raw material provenancing. Isotopic zones for Mesopotamia and Central Asia have been proposed with semi-quantitative characteristic ranges of ε_Nd_ and ^87^Sr/^86^Sr: MP-1 (Mesopotamian Floodplain and Zagros foothill) with a northern sub-zone MP-1N exhibiting slightly less radiogenic Nd isotopic signatures, MP-2 (Iraqi and Syrian deserts/semi-deserts), MP-3 (Syrian Euphrates), CA-1 (Central Asian mountains including the Tian Shan and the Pamirs, and the Amu Darya and Syr Darya), CA-2 (Central Asian deserts and sedimentary basins), and CA-3 (Central Asian loess). For Iran, due to geological diversity and limited data, only a general range has been proposed. Different zones may have overlapping isotopic ranges. The most accurate interpretation requires a combination of chemical and multiple isotopic compositions.^23^ Here, we apply this approach to investigate the provenance of Shadyakh samples by analyzing the Sr, Nd, and Pb isotopes. Figure 5 presents the Sr-Nd isotope data (Figure 5A) and ε_Nd_ vs. 1/Nd plot (Figure 5B) for Shadyakh glass, along with reported TEB glasses.^14^^,^^58^

The ε_Nd_ values of SDK1, 7, 4, 5, and 8 coincide with those of Veh Ardashir glass and fall within the proposed ranges for Iranian and Mesopotamian zones. Northern Mesopotamia’s mountain-derived clastic rocks (MP-1N) were likely the initial source for much of the silica materials used in regional glassmaking,^23^ although the use of western/northwestern Iranian silica sources cannot be ruled out as they would likely bear similar chemical and Nd isotopic signatures indicative of the Zagros geology. At least three distinct sources of silica were used, each with different levels of impurities derived from different erosion or transport conditions: Zagros-A (low-Nd) and Zagros-B1/B2 (high-Nd) (Figure 5B). Zagros-B2 (e.g., SDK4, 5) is higher in Al, Fe, Ti, and Cr than Zagros-B1 (e.g., SDK8). Zagros-B1/B2 silica sources were likely significant in the production of utilitarian glass. Zagros-A silica probably supplied the production of colorless, transparent glass such as Samarra-1 (e.g., SDK7). Glass products made using Zagros-A silica were likely exported to Raqqa and eventually remelted to produce new items.^23^ The ^87^Sr/^86^Sr ratios of SDK4, 7, and 8 indicate the use of TEB (MP-1 or MP-3) or Iranian plant ash. The river valleys in northern Mesopotamia have been proposed as significant suppliers of plant ash.^23^ Therefore, SDK4, 7, and 8 were most likely manufactured using northern Mesopotamian materials and imported from Iraq. Given the compositional similarity between SDK8 and SDK9, it can be assumed that SDK9 shares a similar origin.

SDK1’s ^87^Sr/^86^Sr ratio falls within the ranges of Central Asian deserts and plains (CA-2), western Iraqi and eastern Syrian semi-deserts/deserts (MP-2), or Iran, confirming in any case the use of a different ash source from SDK7. The overlapping bioavailable ^87^Sr/^86^Sr ranges of CA-2/MP-2/Iran are due to commonly occurring Meso-Cenozoic marine sedimentation in these regions, as well as certain surface processes (e.g., sediment weathering, aeolian deposition, and calcite buildup) that are prevalent in these arid environments and affect the bioavailable Sr isotopic composition in soil.^23^ Based on SDK1’s chemical and isotopic compositions, SDK1 could be either an eastern product made using Iranian or Central Asian ash and undetermined low-impurity Iranian silica, or a TEB product made using Zagros-derived (e.g., Zagros-A) silica and MP-2 plant ash. Considering the low Cr/La ratio of SDK1 (a signature of eastern silica, Figure 7), we favor the former possibility. This silica source, possibly from eastern Iran or nearby, would distinctly exhibit very low REE, low Cr, and moderate Al, Ti, and Fe levels. The presence of quartz deposits in this region, such as those near Mashhad, suggests the availability of potential glassmaking materials. If our suggestion for SDK1 holds, Iran was able to produce colorless glass, which would share many of the low-impurity characteristics with Samarra glass but can be distinguished by Cr/La and ^87^Sr/^86^Sr ratios. Note that the few Veh Ardashir samples with ^87^Sr/^86^Sr ratios comparable to that of SDK1 (Figure 5A) have rather high Cr/La ratios. It should be emphasized that one sample is insufficient to establish the Iranian production of low-impurity glass, and SDK1 may well be an outlier of TEB glass. More data are needed to confirm the independent production of colorless glass in this region.

SDK5’s ^87^Sr/^86^Sr is lower than typical TEB/Iranian ranges, suggesting a unique plant ash source. This external source was from inland (^87^Sr/^86^Sr much lower than seawater) and unlikely from areas with extensive Meso-Cenozoic marine sedimentation. A previously analyzed Nishapur sample also displayed a relatively low ^87^Sr/^86^Sr of 0.708124 (Table S3).^38^ One plausible origin of this ash source is inland Egypt, e.g., along the Nile. SDK5 shows comparable P and B levels to Egyptian glass. Late Bronze Age Egyptian plant-ash glass has a ^87^Sr/^86^Sr range of 0.70780–0.70817.^25^^,^^60^^,^^61^ Alternatively, based on similar ^87^Sr/^86^Sr values and P levels, this ash source may be connected to the ash used for Banias glass (median ^87^Sr/^86^Sr = 0.70772^17^). Nonetheless, SDK5’s MgO/CaO ratio differs from that of Egyptian or Banias glass (Figure 4B). In any case, SDK5 was likely produced using Zagros-derived silica (indicated by ε_Nd_) and an external ash source. The use of externally sourced ash, as observed in SDK5, may have been necessary for glassmaking if there were no suitable plants growing locally. This underscores the need to consider the origins of ash and silica separately.

SDK6 is likely a product of Raqqa. Both Sr and Nd isotope values for SDK6 are close to Raqqa raw glass, which was likely made using low-ε_Nd_ semi-desert sand in Syria (MP-2) and ash from the TEB (MP-1/MP-3).^14^^,^^23^ Its chemical signature is also commensurate with Raqqa glass. Possible links between some glass from Nishapur and Raqqa glass have been noted before based on trace elements associated with the silica used.^11^

The ε_Nd_ values of SDK2 and 10 fall into the estimated range of Central Asian deserts and sedimentary basins (CA-2). SDK2 shows a high ^87^Sr/^86^Sr ratio within the range of Central Asian mountains and the Amu/Syr Darya (CA-1), further confirming its Central Asian origin. SDK3 displays a relatively low ε_Nd_ value, pointing to a silica source associated with geologically older formations, with Central Asian mountain-derived sediments (CA-1) being one of the possibilities. However, SDK3’s ε_Nd_ also lies on the less radiogenic end of MP-1N, and the possibility that SDK3 is from recycled glass should be considered (see in the following). The relatively low ^87^Sr/^86^Sr ratios of SDK3 and 10 are consistent with the ranges of Iran and the TEB (the latter is less likely due to the distance). As discussed later, the Khorasan region may partially overlap in the bioavailable ^87^Sr/^86^Sr range with the rest of Iran. A combination of Iranian/Khorasan ash with Central Asian silica materials at regional glassmaking sites was logistically plausible.

Glass mixing can shift the isotopic compositions of glass products toward the average isotopic signature of regionally circulated glass. The relationship between two distinct endmembers in glass mixing can be inferred from a linear trend in the ε_Nd_ vs. 1/Nd graph.^23^ In Figure 5B, we have included three samples SLC22, 23, and 12 from San Lorenzo, Italy,^59^ which were suggested as likely originating from Central Asia based on Sr-Nd isotopes.^23^ These samples, along with SDK2, 3, and 10 (highlighted in orange), may tentatively suggest a mixing line between impure sands sourced from Central Asian deserts/basins and cleaner Iranian/Mesopotamian silica Zagros-B, though more isotopic results are needed to test this further. If this interpretation is correct, SDK2, 3, and 10 or their precursors were likely produced using Central Asian sands and then traded to and used in Iran before being remelted at the end of their use life. SDK3 (with a Nd profile similar to SLC12) may have undergone particularly extensive recycling/mixing, leading to intermediate Cr, Cr/La, Nd, and ε_Nd_ values between typical ranges of Central Asian and Zagros silica materials. It is possible that making bulky products such as SDK3 required a large amount of glass, making the use of mixed glasses more probable. The approximate range for the silica source(s) utilized in Central Asian glassmaking is denoted with an orange circle in Figure 5B. Our proposal for the raw ingredient origins of Shadyakh samples is summarized in Table 1. The disentanglement of ash and silica for “two-dimensional provenance” can provide higher-resolution insights into production technology and trade in comparison to the grouping of samples based on chemical composition only.

**Table 1:** Proposed origins for the raw materials used to make Shadyakh samples

	Tigris-Euphrates ash 1	Tigris-Euphrates ash 2	Tigris-Euphrates ash 3	Iranian/Khorasan ash	Central Asian desert ash	Central Asian rivers ash	Other ash
Zagros silica							
Zagros-A	SDK7 *(∼Samarra)*						
Zagros-B1	SDK8, 9 *(∼Samarra)*						
Zagros-B2		SDK4					SDK5
Mesopotamian desert silica			SDK6 *(∼Raqqa)*				
Central Asian silica (desert/basin)				SDK10, SDK3 (mixed)		SDK2	
Iranian silica (low-impurity)				SDK1			

None of the Shadyakh samples contain sufficient amounts of lead that would signify the intentional addition of lead. The Pb isotopes in SDK2, 3, and 10, which are samples likely made from remelted glass, may be related to colorants (probably Cu-based) present in the original glass. It has been pointed out that, for Europe and the Mediterranean, the Mesozoic and Cenozoic geology associated with the Alpine orogen may not give rise to sufficient variations in Pb isotopes, thus limiting their usefulness as a means of determining provenance.^62^ This limitation also applies to Mesopotamia and Iran in general. Indeed, the Pb isotopic compositions of the Shadyakh samples are largely similar, except for SDK10, whose ^208^Pb/^204^Pb ratio is much lower than others. Within surrounding regions in Asia, such a distinctly low ratio has only been observed in geologically old formations in northern China (North China Craton), southwestern China (South China Craton), central China (Qinling-Dabie Orogen), northern India and Pakistan (Indian Shield), as well as old outcrops in the Central Asian Orogenic Belt.^62^ This suggests that the Pb content in SDK10 may have originated from a minor portion in the original glass melt, which was probably East/South/Central Asian glass containing a significant level of lead with a low ^208^Pb/^204^Pb ratio.

### Organization of glass production

The study of Shadyakh samples reveals the use of various silica and ash sources to make Islamic plant-ash glass. Islamic glassmakers may have, within technological and logistical constraints, used regional ash and silica sources in flexible combinations, resulting in compositional variations and overlaps. This could have taken place with smaller, nearby glassmakers sharing some of the raw materials, or with a glassmaker having multiple raw material suppliers. The combination of one ash/silica source with different silica/ash sources has been observed in Sasanian glass from Veh Ardashir.^32^^,^^50^^,^^51^,^58^ With multiple combinations of raw materials, producer-specific compositional signatures could have become somewhat blurred. This might have occurred for Iranian glass. The reason to use alternative raw materials could be a shortage or high cost of certain raw materials (for instance, when making cost-sensitive utilitarian glass). The diversity of raw material sources used for glassmaking may challenge the notion of a singular production recipe associated with a specific site, which seems to contrast with the relatively more site-specific compositions for early Islamic glass from further west. This does not invalidate the use of regional compositional signatures, as glass production was likely regionally organized: if raw materials were limited to a number of regular sources from the same region, their combinations may still lead to relatively stable glass compositions (although with variations), exhibiting regional signatures corresponding to large-scale geo-environmental contexts. In most Shadyakh samples, the origins of silica and ash within each sample align geographically (e.g., SDK4, 7, 8, 9, 6, 2), indicating that glassmakers for each of these objects used ash and silica sources within short distances. The preference for nearby or local materials can be attributed to lower logistical costs, a local supply network, and familiarity with the materials. Certain regional materials likely played a significant role. For instance, Iraq may have relied on Zagros silica and northern Mesopotamian ash, while Iranian ash and Central Asian desert sands were probably significant in regions east of the Zagros, although material supplies could have varied over time. In light of this, we can identify “production zones” based on supply networks that internally share raw materials for glassmaking. For example, using different TEB sources of raw materials for TEB glassmaking led to the unique but internally diverse compositions of TEB glass. This may particularly benefit the analysis of Iranian and Central Asian glasses where glass production appears to have been more decentralized, requiring an organization model different from, e.g., the mass-produced early Islamic glass in the Abbasid caliphate.

Trade was closely associated with plant-ash glass production, from the procurement of raw materials through the supply of cullet and potentially ingots, to the distribution of finished products. Other than raw materials from regional supply networks, external raw materials (mostly plant ash) were also traded, possibly to meet the need for specialist glass production. Historical records mention the medieval trade of plant ash as a commodity,^63^ although glassmaking was not its only use. Levantine plant ash was once the dominant source of ash used for Venetian glassmaking.^64^ Imported plant ash was used to produce some glass from Raqqa and the Levant.^14^ In addition, raw glass was traded to supply workshops, evidenced by glass cullet and chunks widely discovered, including those reported from Iranian glassworking sites (Section Nishapur and Iranian plant-ash glass).

Mixing and recycling were likely another cause for the observed compositional variations. Although the mixing of glasses from different regions^23^ and the mixing of glasses of different colors have been identified by chemical/isotopic compositions, mixing of similar glasses, for example, colorless glass from the same region, also likely occurred. Similar to regional primary glassmaking, mixing or recycling was likely also a mostly regional phenomenon. If most of the glasses to be mixed were originally made using raw materials from the same region, the regional compositional signatures would be retained in the resulting mix, although any producer-specific signature would be lost. The characteristics of raw materials proposed by glass provenance may be understood as the regional average for raw materials used to make the melted precursors.

**Figure 6:**
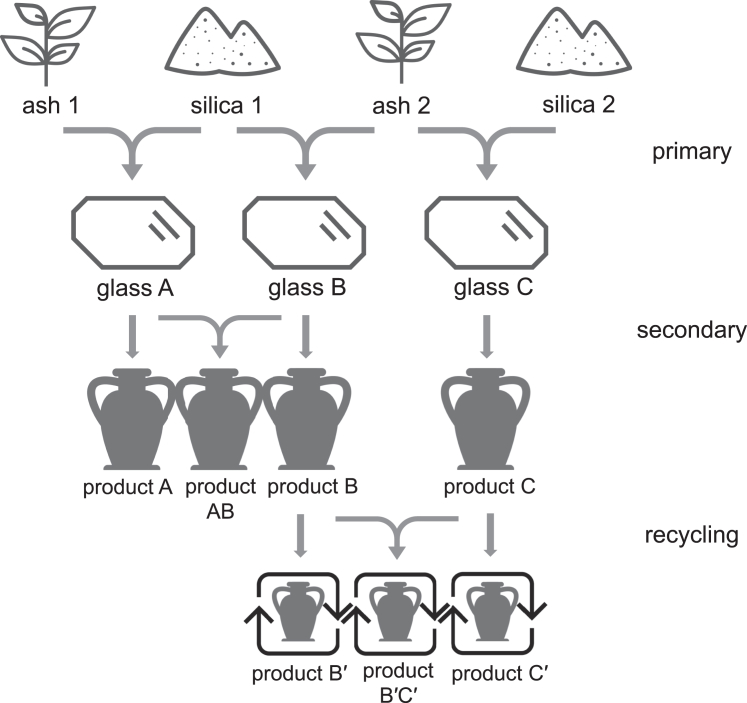
An illustration of possible organization of Islamic plant-ash glass production

The organization of the glass industry reflects the social infrastructure underpinning glassmaking. For instance, natron glass production during Antiquity likely involved primary factories and secondary workshops, and natron glass compositions should resemble primary raw glass (and the regional sands used in primary factories) rather than displaying secondary-workshop-specific fingerprints, and natron glass mixing and recycling could also have occurred in workshops.^65^ Here, Figure 6 schematically suggests how the Islamic plant-ash glass industry was likely organized, taking the diverse sources of raw materials and the potential occurrence of mixing into consideration. Primary glassmaking involved the use of different ash and silica associations (Ash/Silica 1 and 2), leading to the production of various raw glass types (Glasses A, B, C). These primary raw glasses were subsequently used to produce final products (Products A, B, C) either at the same location as primary glassmaking or at separate locations. A mix of raw glasses made using the same silica, likely produced in nearby locations, would retain the “silica signature” (e.g., Product AB, from Glasses A and B) if it occurs. Some glass products underwent recycling to produce new items (e.g., Products B′, C′, or in the case of mixing glass at recycling, Product B′C′). The possibility of mixing primary and recycled glasses (e.g., Product BC′ from Glass B and Product C′ if it occurs) is not represented. In principle, Nd isotope mixing lines may help identify mixed glass derived from precursors made using different silica materials (e.g., Product B′C′). Similarly, Sr isotope mixing lines could assist in identifying mixed glass resulting from precursors made using different ashes (e.g., Product AB), provided there is sufficient contrast in the isotopic signatures of raw materials. However, the extensive overlaps in ^87^Sr/^86^Sr ratios and potentially large variability in ash compositions may limit the application of Sr isotope mixing lines.

### Nishapur and Iranian plant-ash glass

Nishapur was one of the most significant findspots for Islamic glass in the last century. Excavators have inferred the presence of a sophisticated early Islamic glass industry in the city based on the abundance and high quality of glass vessels.^30^^,^^42^ The glass finds in Nishapur have been categorized into colored and colorless groups, with the latter containing generally lower levels of impurities.^66^ However, color (or the absence thereof) is not a reliable indicator for technological divisions. Based on the Met’s Nishapur collection, Wypyski suggested that locally produced glass was more likely represented by simple, colored vessels with high levels of Ti, Zr, and Cr (“Type B” in his grouping) if Nishapur indeed had an Islamic glass industry.^30^ The diverse compositions of Nishapur glass have also led to speculation that the primary production of Islamic glass may not have taken place in Nishapur.^32^

Melted glass discovered in the Shadyakh furnace suggests that glassworking occurred there. Future analysis of the furnace, melted glass, and possible glass waste at Shadyakh may further elucidate the nature of this facility. Previous research has examined two glass ingots from Nishapur: one belongs to Wypyski’s Type A (low impurities, mainly wheel-cut or undecorated colorless glass) and the other to Type B.^30^ Nevertheless, the possibility of primary glassmaking in the city cannot be ruled out at this time, considering the presence of unanalyzed evidence that is potentially related to production.

On a broader scale, it is unlikely that Iran, which had strong connections with the glassmaking region of TEB, completely lacked primary production of Islamic glass. Current direct evidence for Iranian glassmaking is insufficient. In addition to the furnaces in Nishapur excavated in recent decades,^44^ glass furnaces were also discovered in the 1970s in Sirjan by Andrew Williamson^42^ and in Gorgan by Mohammad Kiani,^35^ and remains potentially related to Islamic glass production have been found in recent surface surveys in Old Sirjan City.^67^ Further analysis of these findings is necessary to identify evidence for primary production, in addition to that for glassworking. Glass chunks, cullet, or waste indicating glassworking have also been reported in Siraf,^34^ Nishapur,^30^ and Gorgan.^32^

Distinct compositional signatures of glass may imply the existence of local primary glassmaking. Unique compositional subtypes of glass from Siraf and the prominence of a specific glass subtype in Gorgan have led to conjectures about primary production.^32^^,^^34^ Nonetheless, glass from Iran often exhibits varying chemical or isotopic compositions that overlap with those of TEB or Central Asian glass, reflecting the geological and environmental continuity of the landscapes. For instance, due to the dominance of Zagros geology, glasses produced in the TEB and western Iran (if it occurred there) may be compositionally and/or isotopically similar. In this case, it is also possible that some Iranian samples previously classified as TEB products were actually manufactured in Iran.

**Figure 7:**
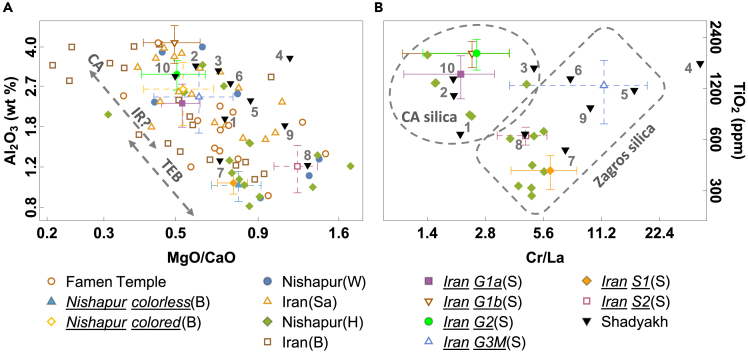
(Color online): Chemical relations of Islamic plant-ash glasses from or potentially related to Iran The data include Nishapur and other Iranian cities,^11^^,^^30^^,^^32^^,^^35^^,^^37^^,^^66^ and Famen Temple, China.^68^ The data for Nishapur colorless/colored glass (B) are from Lankton’s reanalysis using LA-ICP-MS.^15^ Data for Iranian glass (B),^37^ Iranian glass (S),^32^ and Iranian glass (Sa)^35^ comprise artifacts from Hamadan, Qom, Ray, Gorgan, Nishapur, Kangavar, Takht-i-Sulaiman, Susa, and Ghubayra. Panel (A): These glasses likely consist of glass produced in Central Asia (CA), Iran (IR), and the Tigris-Euphrates Basin (TEB). Panel (B): The approximate ranges of Central-Asian-type and Zagros-type silica materials are indicated. Symbols in the legend simply correspond to the grouping in original publications. The analytical methods used to obtain these data and the number of elements reported vary among these samples.

Figure 7 displays the compositions of Islamic glass from Iranian cities.^11^^,^^30^^,^^32^^,^^35^^,^^37^^,^^66^ Islamic glass from Famen Temple, Shaanxi, China, dated to the Tang Dynasty^68^ is also included for comparison. In Figure 7A, Al_2_O_3_ tends to increase while MgO/CaO decreases as the origin of glass shifts from west to east.^15^^,^^34^ Central Asian and TEB glasses exhibit high Al_2_O_3_, low MgO/CaO and low Al_2_O_3_, high MgO/CaO signatures, respectively, with potentially Iranian products expected to exhibit Al_2_O_3_ and MgO/CaO values between those of TEB and Central Asian glasses. However, the defining compositional characteristics of Iranian glass remain elusive and may overlap with glasses from neighboring regions. Figure 7B shows a low Cr/La group that used Central-Asian-type silica and a high Cr/La group that utilized Zagros-type silica. These silica-specific provenances for Iranian glass are partially due to glass traded into Iran, but may also be partially explained by Iranian silica materials that have similar geological origins with those from neighboring regions.

Evident in the origins of Shadyakh samples, manufactured glass products flowed both eastward and westward in long-distance trade across regions, suggesting that utilitarian glass was traded across long distances. Figure 7A reveals that, within many of the analyzed assemblages from the eastern Silk Roads, glasses with compositional signatures of Central Asia, the TEB, and possibly Iran are often found *in tandem*. Glasses belonging to Central Asian, TEB, and potentially Iranian types were found in both Nishapur and Merv—although their proportions in each city differ.^69^ It can be postulated that the sites sharing similar technological types of glass likely participated in a common trade network. For example, the renowned assemblage from Famen Temple consists of some of the best-preserved early Islamic glass vessels from a secure archaeological context. Previous scientific studies have proposed links between Famen Temple glass and glass from Nishapur and Syria.^66^^,^^68^ Vessels visually similar to those in Famen Temple have been found in Iran (e.g., Nishapur) and Iraq (e.g., Samarra)^6^^,^^42^^,^^70^ (also see the British Museum's Samarra glass collection). In Figure 7A, the great majority of Famen Temple vessels indeed fall into the range for Iranian glass or the overlapped range for Iranian and TEB glasses, although a few samples seem to have Al_2_O_3_ and MgO/CaO aligned exclusively with either Central Asian or TEB types. It is plausible that trade hubs such as Nishapur and other major metropolises, where various types of glass were gathered, traded, and sometimes reworked, facilitated the “mix and match” of diverse glasses.

### Central Asian plant-ash glass

Because historical cultures transcended modern geopolitical boundaries, the regions of Khorasan, Afghanistan, and Xinjiang need to be included in studies on ancient Central Asian glass due to their extensive cultural and demographic association with Central Asia. Regional primary production of Central Asian glass is indicated by distinct chemical and isotopic signatures. While some early glass exhibits distinct compositions (e.g.^37^^,^^71^^,^^72^). implying possible early glassmaking in Central Asia, the majority of data with contextual and chronological information pertain to glass dating to after the mid-1st millennium CE (e.g.^29^^,^^31^^,^^33^). Wypyski suggested that Type C of Nishapur glass, mostly simple vessels or beads high in K/Mg, Al, P, and Y, was linked to Central Asia.^30^

**Figure 8:**
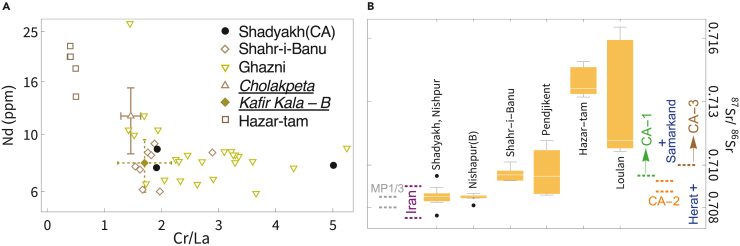
(Color online): Central Asian glass exhibits distinct compositional and isotopic signatures These signatures include (A) relatively low Cr/La ratios and high Nd levels^31^^,^^33^^,^^38^ and (B) relatively high ^87^Sr/^86^Sr ratios. In Panel (B), the Nishapur glass data are from the current study (Shadyakh) and from Brill and Stapleton, 2012^38^ (indicated as “B”, Table S3). Available Sr isotope data for Samarkand and Herat alkali and plant-ash glass from Silk Road regions^38^ (Tables S4 and S5) and Sr isotope baselines for Iran, MP-1, MP-3, CA-1, CA-2, and CA-3 ^23^ are included for comparison.

Previously, high potash levels (K_2_O > 4%–4.5%) were considered a key characteristic of Central Asian glass.^72^^,^^73^ However, this is not a universal feature of Central Asian glass since a large portion of glass from Fergana Valley, Ghazni, and Kafir Kala contains K_2_O levels between 2% and 4%.^29^^,^^31^^,^^33^^,^^71^^,^^74^ Meanwhile, a moderate to high level of Al_2_O_3_^73^ is observed for the majority of Central Asian plant-ash glass. Relatively juvenile felsic rocks (such as granite) are abundant in the larger Central Asian Orogenic Belt,^23^^,^^75^ which are rich in feldspar and typically contain elevated levels of Al, Na, and K. Thus, part of the Al, Na, and K contents in Central Asian glass likely derive from felsic rocks that constitute the sources of Central Asian silica, in contrast to some of the Zagros-derived silica materials deriving from (ultra)mafic rocks. The Al contents are usually associated with the presence of rock-forming minerals such as feldspar and pyroxene. These minerals, as well as accessory minerals such as monazite, allanite, titanite, and zircon, also give rise to elevated levels of heavy elements and REEs. Central Asian glass typically contains Nd > 6 ppm (Figure 8A) and high levels of many other light REEs, which are significantly higher than most glasses made using Zagros-type silica materials. Another distinguishing characteristic is the low Cr content or a low Cr/La ratio (Figure 8A) (Cr/La <5^69^), and glass from Hazar-tam (near Kashgar, Xinjiang) even displays Cr/La ratios under 1.^38^ Compared to Nishapur, glass found in Merv comprises a higher percentage of high-Al and low-Cr groups and a lower portion of TEB-type glass,^69^ which seems to be consistent with its closer location to the heartland of Central Asia.

Central Asian glass tends to display relatively high ^87^Sr/^86^Sr ratios (>0.7087), as observed in glasses from Khorasan (Shahir-i-Banu), Transoxiana (Pendjikent), and western and eastern Tarim in Xinjiang (Hazar-tam and Loulan) (Figure 8B). These samples exhibit significantly higher ^87^Sr/^86^Sr ratios than most glasses from the TEB and Iran. Central Asian glass may have a moderate to low ε_Nd_ value, e.g., ranging from −8 to −4 (Figure 5B), potentially with glass mixing, but Nd isotopic data are currently still very limited. The use of mountain-derived silica in Central Asia for glassmaking, if it occurred, may be supported by future reports of Central Asian glass with very low ε_Nd_ values. In summary, Central Asian glass can be characterized by the following criteria: ^87^Sr/^86^Sr > 0.7087, Al_2_O_3_ > 2.5%, Nd > 6 ppm, Cr/La <5, and probably TiO_2_ > 0.1% (Figures 4, 5, and 8).

Glasses made using materials from different regions in/near Central Asia may still exhibit differences in their chemical/isotopic compositions. Khorasan could potentially access materials in both Iran and Central Asia. The overlap between the Iranian and CA-2 bioavailable ^87^Sr/^86^Sr ranges could reflect Khorasan’s landscape as an intermediate zone between its neighbors. It has been suggested that southwestern Central Asia may have lower bioavailable ^87^Sr/^86^Sr ratios than the northern and eastern parts of Central Asia.^23^ It is possible that significant portions of the Khorasan region may show a bioavailable ^87^Sr/^86^Sr range largely equivalent to the rest of Iran. However, the Amu Darya floodplain and the mountains of Afghanistan may exhibit higher ^87^Sr/^86^Sr ratios due to the erosion of mountain-derived sediments. Consequently, glass manufactured in Khorasan might show ^87^Sr/^86^Sr ratios lower than glass made in areas north of the Amu Darya. This could explain the low ^87^Sr/^86^Sr ratios in SDK10 and SDK3. Greater Khorasan as a cultural unit during the early Islamic period experienced prosperity in crafts-making and trade. A large number of Islamic glass artifacts have been discovered in metropolises in Khorasan such as Nishapur, Merv, and Herat. A wheel-cut bowl found in Venice and dated to around 1000 CE bears the inscription “Khurasan.”^42^ Furthermore, glass used in Iran that exhibits Central-Asian-type composition (e.g., SDK2, 3, 10) may have been produced from remelted glass in Khorasan workshops such as the one in Shadyakh, since Khorasan metropolises were the gateway for Central Asian goods traded to Iran and further west. However, the missing piece of the jigsaw is evidence for primary production. Further studies are necessary to understand Khorasan’s technological connections with the rest of Central Asia and Iran.

### Conclusion

This study presents a highly diverse picture of plant-ash glass production in Islamic West and Central Asia, revealing distinct regional characteristics while emphasizing strong connections among different regions. Through the chemical and isotopic analyses of the Shadyakh samples, we have revealed the diverse origins of Nishapur glass. The glasses from Shadyakh were likely made using raw materials from the TEB, Central Asia, and potentially Iran. Sr-Nd isotopes are shown to be valuable tools for sourcing raw glassmaking materials. Using the integrative chemical-isotopic approach, we have provenanced silica and ash separately and have described the characteristics of Zagros-derived and Central-Asian-type silica. Drawing from the Shadyakh case, we have suggested that the Islamic plant-ash glass industry likely operated through a network of interconnected suppliers and workshops, utilizing multiple sources of raw materials and engaging in secondary production and recycling. While regional compositional signatures are often still recognizable, with decentralized production and the diversity of raw material supplies, compositional variations in plant-ash glass may blur site-specific signatures and add challenges to glass provenance studies. Nonetheless, the variable geochemical signatures of plant-ash glass also present opportunities for renewed investigation, aided by Sr-Nd isotopes, into the organization of glass production. While most raw materials were likely from the regional supply network, inter-regional trade of raw materials also occurred. Furthermore, we have also explicated the situation of glassmaking in Iran and delineated the chemical and isotopic signatures characterizing Central Asian glass.

Our results underscore the importance of Sr-Nd isotopes in determining plant-ash glass provenance. Nd isotopes can be used to trace the origin of silica materials, and more Nd isotopic data reported in the future may greatly benefit the investigation of ancient glass. Importantly, analysis of industrial evidence for primary production is needed in order to understand glass provenance and technologies in Iran and Central Asia.

### Limitations of the study

This study is based on a sample size of ten. The compositional diversity of the Shadyakh samples suggests that, to a large extent, the types of glass in circulation in Nishapur and Iran are represented. The inclusion of multiple lines of evidence, including isotopic and chemical data, has improved the interpretability of data. However, a larger sample pool would help reduce current uncertainties in provenance. Moreover, overlaps of isotopic and chemical signatures exist among different areas, causing ambiguity in interpretation. Despite a balanced consideration of all available information, such as isotopic baselines and mixing lines, some ambiguity may remain. We provide our best possible interpretation in the hope that future analysis of production-related remains will help illuminate provenance studies of Silk Road glasses.

## STAR★Methods

### Key resources table

**Table:** 

REAGENT or RESOURCE	SOURCE	IDENTIFIER
**Software and algorithms**		

ICPMSDATACAL	Liu et al.^76^	NA
Mathematica	Wolfram Research	https://www.wolfram.com/mathematica/

**Other**		

LA-ICP-MS (Agilent 7700e ICP-MS + GeolasPro)	Agilent Technologies Coherent Inc.	https://www.agilent.cco/Library/brochureb/5990-4025EN.pdf https://www.coherent.com/machines-systems/uv-lasers
MC-ICP-MS (Thermo Scientific Neptune Plus)	Thermo Scientific	https://www.thermoscientific.com/

### Resource availability

#### Lead contact

Further information and requests for resources and reagents should be directed to and will be fulfilled by the lead contact, Qin-Qin Lü (QQL20@cam.ac.uk or qinqinlu@icloud.com).

#### Materials availability

This study did not generate new unique reagents.

#### Data and code availability

• Data reported in this study are all included within this publication (and its supplementary material).
• This paper does not report original code.
• Any additional information required to reanalyze the data reported in this paper is available from the lead contact upon request.

### Experimental model and subject details

#### Archaeological samples

The analyzed assemblage consists of 10 glass fragments excavated from Shadyakh, Nishapur. All but two samples (SDK3, 4, deep green) are translucent. Two are colorless (SDK7, 9). Three are mostly colorless with a yellowish or greenish tinge (SDK1, 6, 10). One is translucent light aqua (SDK8), one is translucent green (SDK2), and one is translucent brown (SDK5). A visual inspection reveals varying degrees of environmental impact. SDK2, 4, 7, 8 are almost free of corrosion. SDK9 displays small ‘droplets’ (crizzling) and tiny cracks, typical of being exposed to humidity. An iridescent layer resulting from weathering is evident on SDK1, 6, 9, 10. For some samples, a considerable portion of the surface is covered by an opaque crust from advanced environmental alteration. Such samples include SDK1 (white crust) and SDK3, 6, 10 (tainted crust), although the thick weathered layer of SDK3 appears to have stabilized.

#### Typology

It is possible to infer some of the original vessel forms and the glassworking or decorative techniques used. The vessels were likely made with either mold-blowing or free-blowing techniques, except for SDK3, which was unlikely made by blowing, and SDK5, which may have been partly blown. Over half of the samples are necks or bases, since the thin bodies of blown vessels are prone to breakages and less likely to survive. SDK1 is likely part of an open vessel such as a bowl, decorated with a vegetal pattern, possibly a leaf motif. The relief decoration is sunken on the vessel’s inside and raised on the outside, probably produced by being blown into a mold. SDK2 has a short and narrow neck, a small mouth, and a flared, fortified rim, suggesting that it is from a flask with a globular body, a common vessel type in Nishapur.^42^ The body displays grooves curved anti-clockwise. SDK3 consists of two parts made from the same primary glass: a thick (1–2 cm), gradually tapering tube, and a thick ring encircling the tube decorated with slanted grooves. Its style is similar to a neck fragment from Nishapur in the Brill Collection, reportedly dated to the 9–10th century.^32^ SDK4 is a thick, convex base of an emerald green bottle with a pontil mark, indicating that it was free-blown. SDK5 is the stem of a goblet with ball-shaped designs. Part of the goblet bowl remains attached. SDK6 is likely part of a flask with intricate raised decoration made from applying hot glass on the remaining neck and shoulder. SDK7 is entirely colorless and has somewhat unrefined line-cut patterns, with a gradual curvature indicating a large vessel size. SDK8 appears to be the flat base of a bowl; no decoration is present. SDK9 is a thick-rimmed, thin-bodied fragment probably from a cosmetic bottle, with red-colored, uneven bands following crizzling ‘droplets’ and likely caused by the burial environment. SDK10 is a nearly colorless long neck with a slight bulge at its base, possibly from a large, undecorated flask, a popular vessel form in the Islamic world. Overall, SDK3 is the thickest, followed by SDK4, then by SDK2, while SDK8, 9, 10 are the thinnest. SDK1, 2, 3, 6, 7 have decorative patterns. The market value of a glass vessel could have been determined by an array of factors, such as the form, size/weight, color, decoration, supply of raw glass/materials, the social function of the vessel, and the labor involved in its manufacture.

### Method details

#### Chemical analysis

Small chips were removed from the glass fragments for Laser Ablation Inductively Coupled Plasma Mass Spectrometry (LA-ICP-MS) and Multicollector-Inductively Coupled Plasma Mass Spectrometer (MC-ICP-MS) analyses. The LA-ICP-MS analyses were conducted at the CAS Key Laboratory of Crust-Mantle Materials and Environments in USTC.^77^ The sample was mounted on the sample holder, which was placed in the ablation cell. An Agilent 7700e ICP-MS instrument was used to record signal intensities along with a GeolasPro ArF 193 nm excimer laser sampling system. The aperture size of the ablation spot was 44 μm. The repetition rate was 10 Hz. The carrier glass was helium, flowing at 900 mL/min. Argon was used as the make-up gas, which was mixed with helium before entering the ICP. We typically applied pre-ablation of 10 s or so to remove weathered surface layers. Each analysis started with a background acquisition of approximately 20 s, contained 40 s laser ablation of the sample for data acquisition, and ended with a duration of 35 s for gas flow washing. The acquired signal was monitored in real time to ensure that no contamination from weathered glass was recorded.^78^ We adopted the calibration procedures described by Liu et al.,^76^ making use of multiple external standards and no internal standard. The calibration protocol obtains the ablation yield correlation factor (AYCF) by normalizing the total of metal oxides to 100%. The analysis sequence began and ended with five reference materials (NIST SRM 610, NIST SRM 612, BHVO-2G, BCR-2G, and BIR-1G). NIST SRM 610 and 612 were analyzed at regular intervals in the sequence. During the analysis, real-time count signals were monitored and elements prone to environmental alteration were assessed, and only intervals corresponding to the original glass were selected for data calibration and any weathered layers were avoided (discussed in Lü et al.^78^). We processed the data with ICPMSDataCal software.^76^ To overcome compositional variation within the glass body, we analyzed multiple spots and calculated the average. Low deviation levels were confirmed for each sample.

#### Isotope analysis

For isotope analysis, visible contamination on the chip surface was cleaned, and the chips were slightly abraded to ensure that any potential corrosion on the surface was avoided. The chips were cleaned with an ultrasonic cleaner and distilled water, and were ground to under 100 mesh using an agate mortar and an agate pestle. Sr-Nd-Pb isotopes were analyzed at the CAS Key Laboratory of Crust-Mantle Materials and Environments in USTC, using a well-established analytical protocol^79^^,^^80^^,^^81^^,^^82^ described as follows: Approximately 70–150 mg of fine sample powder was dissolved in 2.5 mL concentrated HF, 0.2 mL HNO_3_ and HClO_4_ in steel-jacketed Teflon bombs, which were heated in an oven at 190°C for one week. After complete dissolution, each sample was dried at high temperature (fuming HClO_4_) on a hot plate; afterward, the mixture was treated with 14 M HNO_3_, evaporated overnight to dryness, and taken up in 3 M HNO_3_ + 3% m/v H_3_BO_3_. The capsule was resealed and kept on a hot plate at 100°C overnight to prepare for chemical purification by ion-exchange resin. The first stage was separating Sr, Nd, and Pb from the matrix using Eichrom DGA resin (50–100 μm, 2 mL). The major element fraction was eluted and collected using 3 M HNO_3_ + 3% m/v H_3_BO_3_, while Sr and Pb fractions were collected for further purification. Then, the column was rinsed with 12 M HNO_3_ to effectively remove any remaining Ca. Finally, the Nd fraction was eluted with 2 M HCl. In the second stage, the Sr and Pb fractions were further purified by Sr-specific resin (100–150 μm, 0.2 mL) before mass spectrometric measurement.^81^^,^^83^ Sr, Nd, and Pb isotopic ratios were measured using a Thermo Scientific Neptune Plus MC-ICP-MS. Whole procedural blanks were less than 100 pg Sr, 50 pg for Nd, and 50 pg for Pb. The Sr and Nd isotopic ratios were normalized to ^86^Sr/^88^Sr = 0.1194 and ^146^Nd/^144^Nd = 0.7219, respectively, using the exponential law. During data acquisition, standard analyses yielded results of ^87^Sr/^86^Sr = 0.710241 ± 13 (2SD, n = 6) for NBS987, ^143^Nd/^144^Nd = 0.512098 ± 12 (2SD, n = 4) for Jndi-1, and ^206^Pb/^204^Pb = 16.9437 ± 12 (2SD, n = 6), ^207^Pb/^204^Pb = 15.5008 ± 15 (2SD, n = 6), and ^208^Pb/^204^Pb = 36.7267 ± 42 (2SD, n = 6) for NBS981. Additionally, USGS reference material BCR-2 was also processed and yielded 0.705037 ± 12 for ^87^Sr/^86^Sr, 0.512637 ± 7 for ^143^Nd/^144^Nd, 18.7591 ± 8 for ^206^Pb/^204^Pb, 15.6273 ± 8 for ^207^Pb/^204^Pb, and 38.7377 ± 25 for ^208^Pb/^204^Pb, which are identical, within error, to the recommended values.^84^
