# Supplementary material for: Connected in diversity: Isotopic analysis refines provenance for Islamic plant-ash glass from the eastern Silk Roads
Source: iScience. 2023 Nov 14;26(12):108450. doi: 10.1016/j.isci.2023.108450 (PMC10758870; doi:10.1016/j.isci.2023.108450)
Supplement: Document S1. Figures S1, S2, and Tables S1–S5 [file mmc1.pdf]

iScience, Volume *26*

Supplemental information

Connected in diversity: Isotopic analysis refines provenance for Islamic plant-ash glass from the eastern Silk Roads

Qin-Qin Lü, Hassan Basafa, and Julian Henderson

Table S1: Chemical composition of the Shadyakh samples, related to Figures 3–8 and Table 1. The oxides (in bold) are given in weight percentage (wt %) and the other elements in parts per million (ppm).

Sample	SDK1	SDK2	SDK3	SDK4	SDK5	SDK6	SDK7	SDK8	SDK9	SDK10
**SiO_2**	62.89	60.71	60.15	63.75	62.21	63.11	71.78	65.95	66.66	62.02
**Na_2O**	14.99	15.99	18.18	17.40	19.22	16.63	11.82	16.04	15.16	13.92
**MgO**	5.66	5.14	4.81	6.00	4.60	4.62	4.91	6.90	6.16	4.43
**Al_2O_3**	1.95	3.31	3.15	3.58	2.34	2.77	1.28	1.22	1.82	2.98
**CaO**	7.97	8.91	7.10	5.31	5.40	6.25	7.17	5.38	5.65	8.88
**K_2O**	4.73	3.77	3.90	1.85	3.83	3.89	1.97	3.16	2.29	3.93
**Fe_2O_3**	0.52	0.78	1.09	1.62	0.79	0.71	0.38	0.34	0.53	0.83
**P_2O_5**	0.54	0.43	0.46	0.20	0.38	0.30	0.07	0.14	0.11	0.55
**MnO**	0.58	0.65	0.79	0.052	0.86	1.39	0.49	0.68	1.41	1.94
**TiO_2**	0.065	0.11	0.16	0.17	0.12	0.14	0.052	0.065	0.092	0.14
Sample	SDK1	SDK2	SDK3	SDK4	SDK5	SDK6	SDK7	SDK8	SDK9	SDK10
Li	13.26	24.93	15.38	9.89	12.80	9.04	20.90	30.29	22.22	14.43
B	142.39	149.25	141.72	87.61	143.22	133.03	58.00	89.97	81.55	140.90
V	9.70	16.70	22.66	33.25	127.51	44.68	8.63	8.56	29.23	39.96
Cr	8.27	15.62	42.82	198.45	80.44	56.67	21.62	24.34	55.10	18.54
Cu	23.38	434.42	563.43	31.11	19.07	74.26	9.04	19.68	12.50	191.94
Co	1.30	16.12	15.48	9.69	17.65	3.32	3.21	8.38	4.83	2.80
Sb	0.61	1.44	1.85	0.22	0.53	0.10	0.11	0.11	0.08	0.41
Zr	27.76	56.91	105.21	73.25	95.86	165.26	39.09	90.63	93.93	91.32
Zn	36.55	60.38	57.88	21.19	26.82	19.56	14.98	27.37	14.50	106.88
Ni	6.03	7.48	26.46	124.56	53.19	27.40	13.99	11.19	15.97	7.56
Sr	450.38	608.60	527.81	406.04	482.89	628.97	423.85	548.33	507.87	655.06
Sn	2.71	11.08	14.49	2.21	2.17	2.06	1.92	1.89	1.82	2.29
Rb	19.22	23.78	22.92	17.81	18.26	26.40	13.88	13.41	13.20	24.01
Y	3.21	7.47	7.36	5.83	5.45	4.43	2.99	4.23	4.86	6.70
Nb	1.03	1.76	3.01	2.10	1.80	2.04	1.21	1.30	1.80	2.44
Cs	0.405	0.430	0.500	0.329	0.366	0.294	0.216	0.111	0.170	0.484
Ba	132.19	232.86	326.93	446.72	1214.86	505.18	123.44	128.05	307.72	1647.49
La	3.98	8.12	8.55	5.45	4.85	7.33	2.97	5.42	5.62	9.70
Ce	7.96	18.56	16.76	12.30	10.52	14.11	6.33	11.53	11.27	15.81

Pr	0.83	1.92	1.89	1.27	1.05	1.52	0.66	1.20	1.27	1.85
Nd	3.09	8.76	7.58	5.56	4.48	6.21	2.69	4.89	5.28	7.42
Sm	0.61	1.50	1.51	1.06	0.78	1.01	0.46	0.87	0.91	1.38
Eu	0.15	0.36	0.33	0.23	0.18	0.22	0.12	0.22	0.20	0.27
Gd	0.46	1.40	1.23	1.05	0.68	0.84	0.48	0.87	0.80	1.22
Tb	0.08	0.21	0.18	0.14	0.10	0.10	0.07	0.11	0.12	0.17
Dy	0.51	1.35	1.20	0.95	0.75	0.80	0.47	0.77	0.76	1.08
Ho	0.12	0.26	0.23	0.21	0.17	0.16	0.09	0.13	0.15	0.23
Er	0.32	0.68	0.75	0.53	0.55	0.39	0.31	0.39	0.52	0.62
Tm	0.05	0.09	0.11	0.08	0.09	0.07	0.05	0.07	0.08	0.08
Yb	0.36	0.65	0.75	0.71	0.57	0.41	0.34	0.42	0.48	0.49
Lu	0.06	0.10	0.11	0.10	0.09	0.09	0.04	0.07	0.07	0.11
Hf	0.64	1.26	2.29	1.65	2.06	3.54	0.87	2.02	2.08	1.89
Th	1.07	1.97	2.25	1.69	1.86	1.60	0.89	1.30	1.47	2.11
Pb	5.60	130.82	134.75	3.79	9.66	33.20	2.19	4.70	2.79	556.54
U	0.55	0.78	0.71	0.50	0.68	0.77	0.49	0.43	0.53	1.26
MnO/ Fe_2O_3	1.12	0.83	0.73	0.03	1.08	1.97	1.27	2.03	2.68	2.33
K_2O/P_2O_5	8.76	8.78	8.46	9.27	10.17	13.00	26.34	22.61	19.91	7.20
MgO/K_2O	1.20	1.36	1.24	3.25	1.20	1.19	2.49	2.18	2.69	1.13

Table S2: The strontium, neodymium, and lead isotope results of the Shadyakh samples, related to Figures 5, 6, 8 and Table 1.

Sample	$^{87}Sr/^{86}Sr$	2SE	$^{143}Nd/^{144}Nd$	2σ	ε_{Nd}	$^{206}Pb/^{204}Pb$	2σ	$^{207}Pb/^{204}Pb$	2σ	$^{208}Pb/^{204}Pb$	2σ
SDK 1	0.708945	0.000012	0.512291	0.000010	-6.8	18.6153	0.0017	15.6523	0.0017	38.6909	0.0050
SDK 2	0.709516	0.000012	0.512337	0.000010	-5.9	18.9866	0.0006	15.7214	0.0006	39.3744	0.0020
SDK 3	0.708510	0.000014	0.512225	0.000010	-8.1	18.5615	0.0006	15.6817	0.0006	38.7520	0.0018
SDK 4	0.708243	0.000011	0.512279	0.000013	-7.0	18.6729	0.0018	15.6789	0.0019	38.6724	0.0056
SDK 5	0.707643	0.000013	0.512214	0.000018	-8.3	18.7241	0.0013	15.6939	0.0014	38.8466	0.0044
SDK 6	0.708569	0.000013	0.512070	0.000012	-11.1	18.5484	0.0006	15.6551	0.0006	38.6773	0.0018
SDK 7	0.708523	0.000011	0.512342	0.000018	-5.8	18.9395	0.0022	15.6664	0.0023	38.6639	0.0072
SDK 8	0.708306	0.000012	0.512233	0.000017	-7.9	18.7862	0.0013	15.6914	0.0012	38.8391	0.0035
SDK 10	0.708318	0.000011	0.512351	0.000013	-5.6	18.6633	0.0003	15.6301	0.0004	35.6922	0.1085

Tables S1 and S2 are also deposited in Lu, 2023 [1].

For the convenience of readers, Tables S3–S5 provide legacy data from previous Sr isotope analyses in Brill and Stapleton, 2012 [2].

Table S3: Sr isotope data of six plant-ash glass samples from Nishapur [2], related to Figure 8. The compositional type is inferred from comparing major elements data (Si, Al, Mg, Ca, Na, Fe, K) reported in Brill, 1999 [3] and Sr isotope data in Brill and Stapleton, 2012 [2] to known signatures of contemporary glass.

Sample	$^{87}Sr/^{86}Sr$	Type	Comment
1824	0.708579	Samarra-1/Iranian high quality	Colorless glass. Both major element concentrations and Sr isotopic value are similar to SDK7.
5320	0.708437	Samarra-1/Iranian high quality	Colorless glass. Both major element concentrations and Sr isotopic value are similar to SDK7.
5307	0.708531	Tigris-Euphrates Basin/Iran/Khorasan	Chemical and Sr isotopic compositions are within possible ranges of Tigris-Euphrates Basin glass, Iranian glass and Central Asian (Khorasan) glass.
5306	0.708647	Tigris-Euphrates Basin/Iran/Khorasan	Chemical and Sr isotopic compositions are within possible ranges of Tigris-Euphrates Basin glass, Iranian glass and Central Asian (Khorasan) glass.
5308	0.708124	Tigris-Euphrates Basin/Iran	Chemical and Sr isotopic compositions are within possible ranges of Tigris-Euphrates Basin glass and Iranian glass.
5323	0.708559	Samarra-1/Iranian high quality	Colorless glass. Both major element concentrations and Sr isotopic value are similar to SDK7.

Table S4: Sr isotope data of alkali or ash samples from the Silk Road regions [2], related to Figure 8. The notes are from Vol. 1 of the same book series [5].

Sample	Location	$^{87}Sr/^{86}Sr$	Note
1324	Jezaziyat, Iraq	0.708005	ashed desert plant
1326	Baghdad, 1967	0.708128	ashed *souq. Chinan* plant. Stems and pieces. Dried and chopped.
4401	Iraq, 1960s	0.707991	ashed *chinan* plants. Thought to be from Iraq. Stems and small debris. Original label was lost in flood.
1380	Qasr al-Hayr, Sharqi, Syria. 1960s	0.707868	ashed chinan plant, said to be used for making *keli*. Woody stems and foliage.
1382	Qasr al-Hayr, Sharqi, Syria.	0.709603	Plant ash. *Keli* (prepared by local workers from *chinan* plants), "from the mosque". Hard, porous, gray chunks. Said to date from 700.
4586	Qasr al-Hayr, Sharqi, Syria. 2006	0.708131	Plant ash.
4447	Samarkand, Uzbekistan	0.711112	Plant ash. Alkali used by tile glazers. Hard, dk. gray lumps. Final firing at 800°C for 1 hr. to remove unburned carbon.
1331	Herat, Afghanistan	0.708987	Plant ash. *Ishghar* used at glass factory of Fayzullah who said that it "comes from the mountains where it flows out of the fire like water".

Table S5: Sr isotope data of plant-ash soda-lime glass from the Silk Road regions [2], related to Figure 8. Due to the lack of trace element data in general, we have not attempted to provenance these samples. Compositional types given by the original note were according to the Al and K criteria proposed by the original authors to distinguish Central Asian (CA) glass. Chemical data of these samples can be found in existing publications [2-4].

Sample	Location	$^{87}Sr/^{86}Sr$	Original note in Brill and Stapleton, 2012 [2]
6058	Shahr-i-Banu, near Mazar-i-Sharif, Afghanistan	0.710144	vessel. CA (high K).
6061	Shahr-i-Banu	0.709535	vessel. CA (high K).
6062	Shahr-i-Banu	0.709596	vessel. CA (high K).
6063	Shahr-i-Banu	0.709265	vessel. CA (high K mod. Al).
6067	Shahr-i-Banu	0.709264	vessel. CA (high K).
6114	Chakhcharan, Afghanistan	0.709209	bottle, foil dec. CA (high-K) or Sas-Islm.
6119	Duldur-Aqur, Xinjiang, China	0.708652	Sas. cut bowl. Sas-Islm.
6120	Duldur-Aqur	0.711446	CA (high K).
6122	Hazar-tam, near Kashgar, Xinjiang	0.713212	CA (high K, high Al, high REEs, low Cr(?)).
6123	Hazar-tam	0.713616	CA (high K, high Al, high REEs, low Cr).
6125	Hazar-tam	0.714711	CA (high K, high Al, high REEs, low Cr.).
6126	Hazar-tam	0.713461	vessel. CA (high Al).
6127	Hazar-tam	0.714369	vessel. CA (high Al).
6128	Hazar-tam	0.713326	earring. CA (high K high Al, high REEs, low Cr).
6129	Hazar-tam	0.714886	earring. CA (high K high Al, high REEs, low Cr).
6130	Qizil, Xinjiang	0.708377	Sas-Islm.
6253	Pendjikent, Tajikistan	0.711149	CA (high K).
6255	Pendjikent	0.708698	bottle and trailings. High K.
6256	Pendjikent	0.709540?<br>0.710250?	bottle neck. High K, high Al.
6258	Pendjikent	0.708582	Sas. cut glass. 'Sas-Islm'.
6811	Lou-Lan, Xinjiang	0.711078	CA (high K).
6812	Lou-Lan	0.710791	vessel. CA (high K, mod. Al).
6817	Lou-Lan	0.711263	CA (high K, mod. Al).
6818	Lou-Lan	0.710646	CA (high K, mod. Al).
6823	Lou-Lan	0.716521	CA (high K, mod. Al).
6827	Lou-Lan	0.715909	vessel. CA (high K, mod. Al).
8503	Hanguya Tati, Xinjiang (Khotan)	0.715043	vessel. CA (high K high Al).
8505	Togujai, Xinjiang	0.711139	CA (high K high Al).
8506	Kelpin, Xinjiang	0.716646	bead. CA (high K high Al).

Supplemental figures show a possible glass workshop in Trench A-II, Shadyakh, Nishapur.

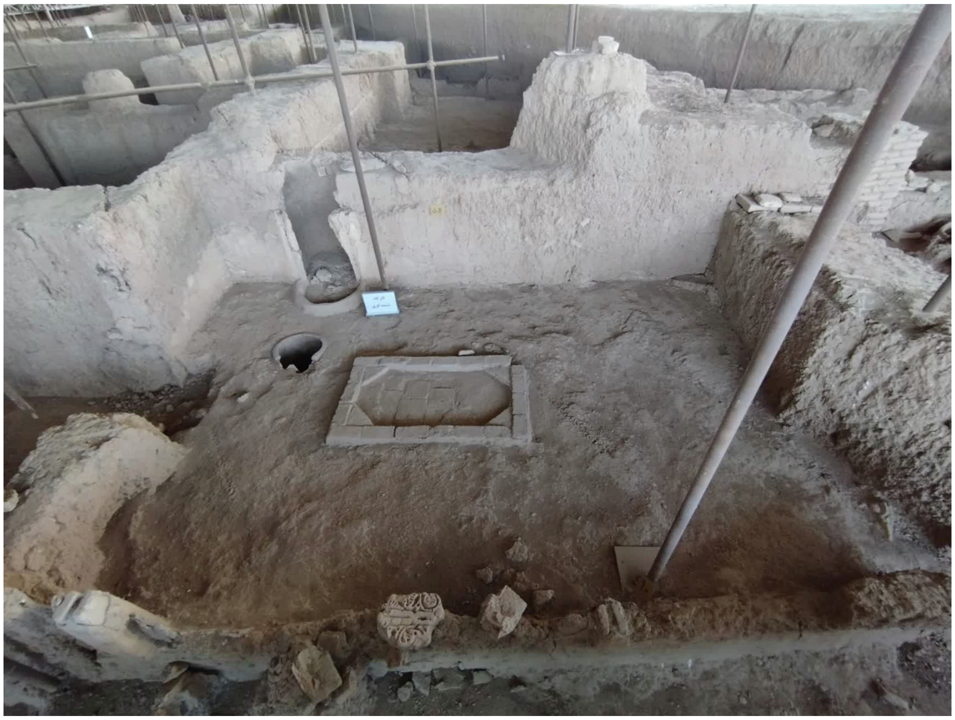

Fig. S1: A possible glass workshop in Trench A-II, Shadyakh, Nishapur, related to Fig. 1.

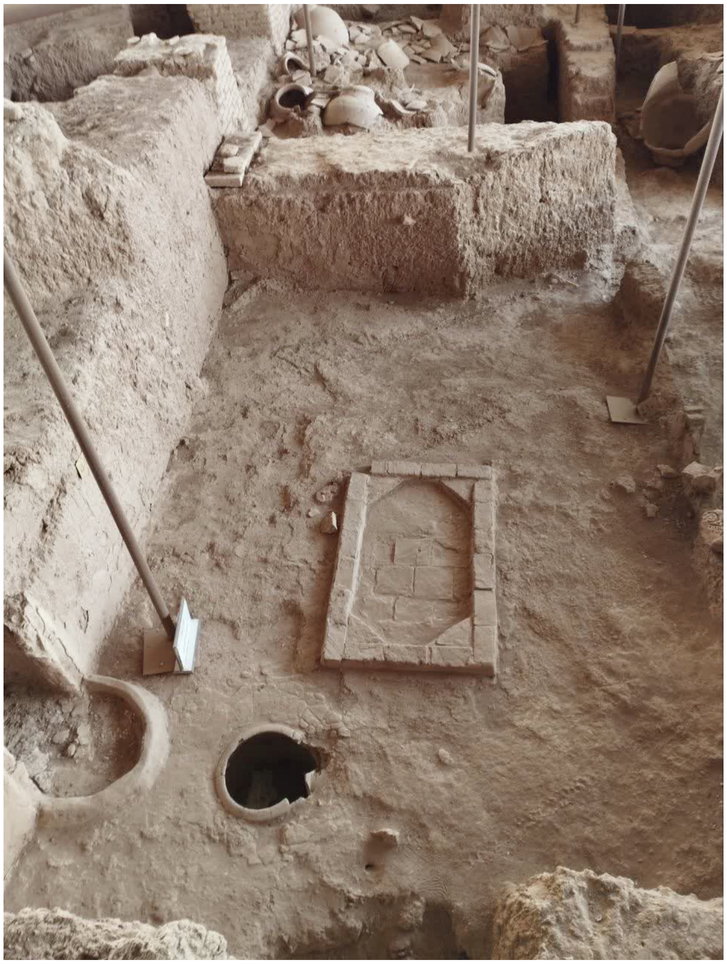

Fig. S2: A possible glass workshop in Trench A-II, Shadyakh, Nishapur, related to Fig. 1.

1. Lu, Q.-Q. (2023). Dataset in: Connected in diversity: isotopic analysis refines provenance for Islamic plant-ash glass from the eastern Silk Roads. Mendeley Data. 10.17632/55znfkp7x6.2
2. Brill, R.H., and Stapleton, C.P. (2012). Chemical Analyses of Early Glasses. Volume 3: The Years 2000–2011, Reports, and Essays (Corning Museum of Glass).
3. Brill, R.H. (1999). Chemical Analyses of Early Glasses: Volume 2: Tables of Analyses (Corning Museum of Glass).
4. Brill, R.H. (2009). Opening remarks and setting the stage: lecture at the 2005 Shanghai International Workshop on the archaeology of glass along the Silk Road. In Ancient glass research along the Silk Road (World Scientific), pp. 109–147.
5. Brill, R.H. (1999). Chemical Analyses of Early Glasses: Volume 1: Catalogue of Samples (Corning Museum of Glass).
